# A hybrid AI approach for supporting clinical diagnosis of attention deficit hyperactivity disorder (ADHD) in adults

**DOI:** 10.1007/s13755-020-00123-7

**Published:** 2020-11-20

**Authors:** Ilias Tachmazidis, Tianhua Chen, Marios Adamou, Grigoris Antoniou

**Affiliations:** 1 School of Computing and Engineering, University of Huddersfield, Huddersfield, UK; 2 South West Yorkshire Partnership NHS Foundation Trust, Wakefield, UK

**Keywords:** AI in medicine, Decision making support, ADHD diagnosis, Machine learning model, Knowledge model

## Abstract

Attention deficit hyperactivity disorder (ADHD) is a neurodevelopmental disorder that includes symptoms such as inattentiveness, hyperactivity and impulsiveness. It is considered as an important public health issue and prevalence of, as well as demand for diagnosis, has increased as awareness of the disease grew over the past years. Supply of specialist medical experts has not kept pace with the increasing demand for assessment, both due to financial pressures on health systems and the difficulty to train new experts, resulting in growing waiting lists. Patients are not being treated quickly enough causing problems in other areas of health systems (e.g. increased GP visits, increased risk of self-harm and accidents) and more broadly (e.g. time off work, relationship problems). Advances in AI make it possible to support the clinical diagnosis of ADHD based on the analysis of relevant data. This paper reports on findings related to the mental health services of a specialist Trust within the UK’s National Health Service (NHS). The analysis studied data of adult patients who underwent diagnosis over the past few years, and developed a hybrid approach, consisting of two different models: a machine learning model obtained by training on data of past cases; and a knowledge model capturing the expertise of medical experts through knowledge engineering. The resulting algorithm has an accuracy of 95% on data currently available, and is currently being tested in a clinical environment.

## Introduction

Attention-deficit/hyperactivity disorder (ADHD) is one of the most common neuropsychiatric conditions with a pooled worldwide prevalence estimated at approximately 5% in school-aged children with persistence of impairing symptoms in adulthood in up to 65% of cases. The pooled estimated prevalence of ADHD in adults is approximately 2.5% [1]. ADHD is characterised by a persistent and impairing pattern of inattention and/or hyperactivity/impulsivity that causes significant impairment across domains [2]. Along with these three main symptomatic clusters, people with ADHD also present with deficits in executive functions, behaviour and emotion regulation, and motivation [3].

For the people who are diagnosed, the modes of interventions for primary ADHD symptoms with robust evidence base are pharmacological and psychological [4]. The first line treatment for adult ADHD is psychostimulants [5]. Medication is safe and effective, with 70% of patients reported improvement compared to 7% of controls [5, 6].

The adverse effects of untreated ADHD are well documented with negative effects on academic outcomes [7–9], social functioning [10], employment [11] but also life itself leading to increased mortality [12]. The total yearly costs to the individual and state combined were recently estimated to be €17,769 per person, per year [13] thus there is strong impetus for interventions.

For the UK, the National Institute for Health and Clinical Excellence (NICE) suggested in 2008 that the standard benchmark rate for referral to a Service in adults is 25 per 100,000 per year [14]. The largest challenge at the moment for the adult population, bearing in mind the relative recency of acceptance amongst the professional community that ADHD can persist into adulthood [15], is the dearth of clinicians appropriately trained and confident to place the diagnosis. Such bottleneck prevents patients receiving appropriate treatments and hence contributes to the morbidity of the adult ADHD.

Recent advance in machine learning has enjoyed a number of successes in medical applications [16–18]. To address this challenge, we wanted to investigate if there was a way by which using clinical information collected from a Service, which delivers a clinical pathway that is compliant with NICE recommendations (i.e. gold standard), can create a decision tool that can automate the process of making a diagnosis. The clinical data collected in this paper is from a NHS specialist mental health provider in the form of screening questionnaires and clinical interviews, which are routinely collected when a new patient is referred. We are not aware of this being achieved anywhere else in mental health populations whereby an AI algorithm will propose diagnostic decisions based on the form of data we use.

Being commonly used in medical settings where the demand of interpretability is generally considered high, knowledge-based systems aim to represent knowledge explicitly via tools such as production or if-then rules, which allow such a system to reason about how it reaches a conclusion and to provide explanation of its reasoning to the user [19]. Following on our previous work [20] where only machine learning-based approaches were used, a new innovative approach is now undertaking that simultaneously employs machine learning and a knowledge-based approach in a hybrid manner.

On the one hand, we trained a prediction model based on machine learning using the data made available to us. The "Experimental evaluation" section demonstrates that by applying machine learning, we can achieve a diagnostic accuracy of 85%. On the other hand, we captured knowledge from medical experts and represented it in the form of rules that may conflict with each other. This model seeks to give yes/no answers for clear-cut cases, while referring the remainder for further assessment by medical experts. The latter outcome reflects the fact that the AI algorithm is meant to serve as a *decision support tool* that increases the productivity of a clinical team, not as a way to reduce or replace the clinical team. We proceeded to combine the two algorithms, with the driving idea that where they are in disagreement, patients are referred to medical experts. On this basis, the algorithm achieves an accuracy of 95%.

The remainder of this paper is organised as follows. "Data collection" section describes the available data. Machine Learning Model" section provides details of the machine learning model, "Knowledge Model" section describes the knowledge model and "Experimental evaluation" section presents experimental results regarding performance. "Conclusions and Future Work" section concludes the paper by presenting current and future work.

## Data collection

A National Health Service specialist mental health provider (South West Yorkshire Partnership NHS Foundation Trust-SWYPFT) made available for analysis all anonymized data for assessments made of ADHD patients in the period between 2014 and 2017. Overall there were 69 such patients. For all these patients, the data contained information which included demographics and a number of validated self-reported screening questionnaires and clinical interviews.

Each patient contains a client ID, which is used to join with other entries related to the patient—see below—and demographic information about age, gender and post code. All this information is in (semi-)structured form. Table 1 summarizes the descriptive statistics of the demographics information. No difference in age was assessed between the two genders through t-test (p-value = 0.06).

**Table 1:** Demographics information

Total number of patients:	69
Age	Average (std)
Whole population	33.01 (9.931)
Men	31.36 (10.85)
Women	36.13 (7.12)

Gender	Number of subjects (%)
Male	45 (65.2%)
Female	24 (34.8%)

Post code	Number of subjects (%)
Wakefield	42 (60.9%)
Barnsley	27 (39.1%)

The screening questionnaires included the Conner’s ADHD Rating Scales [21], the Drug Abuse Screening Test (DAST-10) [22], the Iowa Personality Disorder Screen (IOWA) [23], the Alcohol Use Disorders Identification Test (AUDIT) [24], the Mood Disorder Questionnaire (MDQ) [25], the GAD-7 measuring Generalized Anxiety [26], the Patient Health Questionnaire (PHQ-9) which measures the severity of depression and the HELPS^1^ brain injury screening tool [27]. The clinical interviews were both structured and unstructured. The structured interviews were made using the Diagnostic Interview for ADHD in adults (DIVA) [28] and unstructured where captured in the text of the final medical report which was also provided. In addition, we had data from the scores of the Sainsbury’s Risk Assessment Tool [29] and results from the objective measurement of ADHD symptoms obtained using the QbTest [30].

## Machine learning model

In order to generate an assessment-centered dataset for constructing a predictive model, patient demographics, self-reported assessment, Conner’s Adult ADHD Rating Scale (short version) on the basis of both - self-report and observe mode, the QbTest, and the diagnostic interview for ADHD in Adults are jointed to form the main assessment data. Depending on whether the risk assessment data is combined, two groups of predicted analysis are designed as follows:

- Construct the predictive model by purely using the main assessment data, which consisted of 27 variables. Note that missing values occasionally occur for some of variables. Though more advanced techniques [31] may be employed, this paper adopts the conventional approach that fills with the average value for a continuous variable and mode value for a discrete variable where missing values apply.
- Build the predictive model on the basis of joining the main assessment and the risk assessment data, which results in an additional 66 variables and 93 variables in total. Note that each variable from the risk assessment data is binary that only takes ‘yes’ or ‘no’ for a given assessment question.

Considering the number of available data is relatively small, which only comprises 69 patients in total, the leave-one-out cross validation is utilised such that the learning method is trained on all the data except for one patient and a prediction is made for this particular patient. The performance of a model will be evaluated with accuracy and AUC score. A total number of six popular machine learning algorithms will be employed in an effort to select the best model for clinical use. This includes Support Vector Machine, Naive Bayes, Decision Tree, K-nearest Neighbour, all of which have been considered as top 10 algorithms in data mining [32], as well as Logistic Regression [33] and Random Forest [34].

## Knowledge model

Developing a knowledge model for ADHD diagnosis requires a different approach compared to machine learning. Instead of focusing on a data-centric method, experts in the field of ADHD diagnosis need to be interviewed in order to extract and encode their empirical knowledge. In this work, there was one clinician who is an international expert in adult ADHD. The process was conducted through three interviews. The first interview was to outline the rules, the second to refine them and the third to confirm them. In this way, knowledge that has been acquired through experience (e.g. working with patients) can be transformed into a systematic approach based on if-then rules. More specifically, clinicians have a deep understanding of various tests and questionnaires that need to be conducted, including those mentioned in "Data Collection" section, as well as their meaning and overall contribution towards a diagnosis. However, such experience is not straightforward to encode in a machine readable format while ensuring that the resulting knowledge model leads to diagnosis that follows the rational of a clinical expert.

In order to develop a successful knowledge model, the meaning of each source of data needs to be explored. In general, DIVA scores could be used in order to inform a decision, namely low DIVA scores indicate that ADHD should not be inferred, while high DIVA scores provide a more clear indication towards ADHD diagnosis. However, the impact of each indicator such as DAST-10, IOWA, AUDIT, MDQ, GAD-7, PHQ-9 and HELPS could be used in order to assess the level that patients are affected by substance abuse, personality disorder, alcohol use, bipolar disorder, anxiety, depression and brain injury, respectively. Such indicators need to be considered and weighted towards a decision as well since the presence of high anxiety levels or personality disorder could lead to overlapping symptoms with ADHD.

The unique experience of a clinical expert dictates the contribution of each data source into the decision making process and could be translated into if-then rules such as:

- If DIVA scores are high, then the decision is ‘yes’
- If DIVA scores are low, then the decision is ‘no’
- If multiple indicators are present, then the decision is ‘expert’

The aforementioned rules are not specific and cannot be applied directly on the existing dataset since the definition of high and low DIVA scores is ambiguous. Thus, a threshold based on experience is set in order to differentiate between high and low scores. Moreover, the presence of multiple indicators is independent of DIVA scores. Thus, the decision to refer the patient to a medical expert could potentially override a yes/no decision. Such conflicts can be resolved through rule prioritisation, namely when multiple rules are applicable, the rule with the highest priority is chosen.

The following generic rules were identified and prioritised by the expert:

- Rule #1: If DIVA scores are below threshold, then the decision is ‘no’
- Rule #2: If multiple indicators are present, then the decision is ‘expert’
- Rule #3: If DIVA scores are above threshold, then the decision is ‘yes’

The knowledge model initially considers the applicability of rule #1, where for low DIVA scores the decision is ‘no’. However, in case DIVA scores are high (namely rule #1 is not applicable), the knowledge model needs first to evaluate the presence of multiple indicators. Assuming there are multiple indicators present, both rule #2 and rule #3 are applicable. However, rule #2 has a higher priority, thus the decision is ‘expert’. Note that only when neither rule #1 nor #2 are applicable, the decision is based based on rule #3, namely the decision is ‘yes’.

A comprehensive knowledge model requires a wide range of rules and a carefully selected rule prioritisation. This can be achieved through a trial and error process where clinical experts and knowledge engineers bridge the gap between empirical knowledge and machine readable representation of knowledge. The quality of a developed knowledge model is eventually assessed based on existing data and should be periodically re-evaluated as more data is made available through new patients.

### Hybrid model

The results of a knowledge model could be combined with the results of a machine learning model. Note that the machine learning model provides yes/no answers, while the knowledge model provides yes/no/expert answers. Thus, a hybrid model can be developed by combining the two approaches. When both models agree on a yes/no answer, then this is the final answer. However, when the two models are in disagreement, then patients are referred to a medical expert. Table 2 summarizes all possible outcomes for the hybrid model.

**Table 2:** Possible outcomes of a hybrid model

Machine learning model	Knowledge model	Hybrid model
yes	yes	yes
yes	no	expert
yes	expert	expert
no	yes	expert
no	no	no
no	expert	expert

The hybrid model is thus more robust since a yes/no answer is endorsed by both machine learning and knowledge model. Note that referring patients to medical experts is a valid and desirable outcome since the developed algorithm is designed to speed-up the diagnosis process for clear-cut cases, thus leading to a higher throughput of cases per clinical expert who is already on the team; the aim is not to reduce or replace a clinical team by AI.

Another benefit of the hybrid approach is that the machine learning model provides input even for cases that are referred to clinical specialists - the latter can conduct their evaluation with the additional information that the AI algorithm has a tendency to a particular outcome.

## Experimental evaluation

### Machine learning model

To demonstrate the performance of machine learning algorithms for the predictive modelling of ADHD diagnosis, experiments were conducted using the scikit-learn open source machine learning library for the Python programming language, which integrates the implementation of all aforementioned machine learning approaches with default settings unless otherwise explicitly specified. Performances are reported as accuracy, which is the percentage of correct predictions, i.e. the resultant model predicts positive in case the patient to be diagnosed has ADHD and negative in case the patient does not have ADHD. A perfect classification model would always make correct predictions, resulting in 100% accuracy. In addition, Area Under the Receiver Operating Characteristic (ROC) curve (AUROC or just AUC) is also reported, which illustrates the performance for a binary classification problem, when a threshold is varied on the predictions. AUC is the curve of sensitivity (i.e. true positive rate), plotted against 1-specificity (i.e. false positive rate), which is independent of the prior class distribution, i.e. percentages of positive and negative samples. A perfect classification would produce AUC = 1, while random guessing would produce a AUC = 0.5.

Table 3 summaries the performance on the main assessment report, which consisted of 27 variables. Most algorithms achieve accuracy in the range of 70–80%, with the decision tree algorithm having accomplished the highest accuracy as highlighted in bold, followed by Random Forest and Naive Bayes. In terms of AUC, the three algorithms that achieve the top 3 best accuracies are also competent with each other, resulting in very close AUC. It is worth noting that the experiment at this stage aims to identify the optimum machine learning algorithm for this ADHD predictive modelling task, hence necessary to compare their performances.

**Table 3:** Experimental results on the main assessment data

Machine learning method	# Patients	# Variables	Accuracy(%)	AUC
Support vector machine	69	27	72.464	0.784
Logistic regression	69	27	72.464	0.795
Decision Tree	69	27	**82**.**609**	0.866
K-Nearest neighbour	69	27	59.420	0.558
Random forest	69	27	81.159	0.866
Naive Bayes	69	27	75.362	0.870
Averaged	69	27	73.91±8.30	0.79±0.12

**Table 4:** Experimental results on the main assessment and risk assessment data

Machine learning method	# Patients	# Variables	Accuracy(%)	AUC
Support vector machine	69	93	76.812	0.806
Logistic regression	69	93	75.362	0.815
Decision tree	69	93	**85**.**507**	0.871
K-nearest neighbour	69	93	59.420	0.559
Random forest	69	93	75.362	0.804
Naive Bayes	69	93	72.464	0.740
Averaged	69	93	74.15±8.47	0.77±0.11

In addition to the main assessment data, the experiment carries on utilizing the risk assessment data as well, which evaluates a potential patient historical behaviour that might be related to the occurrence of ADHD. On the basis of the additional 66 variables resulted from the risk assessment, the aforementioned machine learning algorithms construct predictive models joining the main assessment and the risk assessment data, resulting in 93 variables in total.

According to Table 4, in spite of a significantly larger number of variables, performances of the resulting algorithms generally match that of Table 3. That is, most algorithms still perform in the level of 70–80%. However, performances of most algorithms have generally improved (except k-nearest neighbour and Random Forest). This is also expected from a clinical viewpoint, considering a lot of relevant and targeted information is now embedded in the training by utilising the risk assessment which is specifically carried out as a clinical activity. Overall, the decision tree is a clear winner being the only algorithm with accuracy above 80%, as well as top AUC value.

Reflecting on the above two sets of results, decision tree has been the best overall classifier in comparison to five popular alternatives, achieving 2 highest accuracies and 1 top AUC values. In addition, the decision tree algorithm generates a set of if-then rules, with each rule providing a diagnosis specified by the condition. The rule base is interpretable, offering a means to explain how a conclusion is derived, which is necessary for a data-driven model to be employed in practice. In case of rules against medical knowledge, clinicians can easily make changes or simply delete abnormal rules. On the other hand, the risk assessment data has helped improve performance of the decision tree from 82.609 to 85.507% if put into use in conjunction with the main assessment data. Such risk assessment data should be utilised to generate the final model.

### Knowledge model

The knowledge model is based on if-then rules, encoding the knowledge of medical experts. In addition, the best performing machine learning algorithm, namely the decision tree algorithm, generates a set of if-then rules as well. The results of the two models are combined by the hybrid model as described in "Hybrid Model" section, leading to an overall prediction of an ADHD diagnosis. We evaluated all three models over the existing dataset for the 69 patients and compared the results to the diagnosis made by the medical experts. Note that the knowledge model, the machine learning model and the hybrid model are referred below as *KR*, *ML* and *Hybrid* models, respectively.

**Table 5:** Number of patients assigned to each outcome

Model	Yes (%)	No (%)	Expert (%)
KR	14 (20.29)	24 (34.78)	31 (44.93)
ML	39 (56.52)	30 (43.48)	0 (0)
Hybrid	12 (17.39)	23 (33.33)	34 (49.28)

**Table 6:** Number of misclassified patients for each outcome

Model	Yes (%)	NO (%)	Expert (%)
KR	2 (14.29)	1 (4.17)	0 (0)
ML	4 (10.26)	4 (13.33)	0 (0)
Hybrid	2 (16.67)	1 (4.35)	0 (0)

Table 5 shows how patients were classified by the three models. It is evident that in the *ML* model all patients are classified as either having ADHD or not having ADHD (YES/NO outcomes only), while in the *KR* model only approximately 55% of patients are classified to a YES/NO outcome with almost 45% of patients being referred to a medical expert. This is tern leads the *Hybrid* model to classify 50% of patients to a YES/NO outcome and 50% of patients being referred to a medical expert. In general, it is expected that the *Hybrid* model will classify the minimum number of patients to a YES/NO outcome (as both *KR* and *ML* models must provide the same classification) and the maximum number of patients will be referred to a medical expert (those referred by the *KR* model as well as all outcome disagreements between *KR* and *ML* models). It is worth pointing out that a rate of 50% of patients being referred to a senior clinical specialist will significantly speed-up the diagnosis process, considering the fact that currently all patients are assessed by a senior clinical specialist.

Table 6 provides the number of patients whose outcomes were misclassified as well as the corresponding misclassification rate. The *ML* model misclassified 4 (out of 39) patients as having ADHD and 4 (out of 30) patients as not having ADHD. However, since in the *ML* model all patients are assigned to a YES/NO outcome the misclassification rates are relatively low, namely 10.26 and 13.33% for YES and NO outcomes, respectively. Both *KR* and *Hybrid* models exhibit the same number of misclassified patients (two patients as having ADHD and one patient as not having ADHD). However, the *Hybrid* model shows a higher misclassification rate compared to the *KR* model. This is attributed to the fact that the same 3 patients were misclassified by both *KR* and *Hybrid* models, while 3 correctly classified patients by the *KR* model were referred to a medical expert by the *Hybrid* model due to conflicting classifications between the *KR* and *ML* models (for more details, see Table 8). Note that a referral to a medical expert is considered a valid outcome. Nonetheless, it reduces the number of patients assigned to a YES/NO outcome.

**Table 7:** Accuracy of each model per set of outcomes

Model	Yes/No (%)	Yes/No/Expert (%)
KR	**35/38 (92.1)**	**66/69 (95.7)**
ML	61/69 (88.4)	61/69 (88.4)
Hybrid	32/35 (91.4)	**66/69 (95.7)**

**Table 8:** Detailed outcomes of misclassified patients

Medical expert	KR	ML	Hybrid
yes	yes	**no**	expert
yes	yes	**no**	expert
no	**yes**	**yes**	**yes**
yes	expert	**no**	expert
no	no	**yes**	expert
no	**yes**	**yes**	**yes**
no	expert	**yes**	expert
yes	**no**	**no**	**no**

Table 7 presents the accuracy of each model, namely how many patients where correctly classified out of all patients assigned to a specific set of outcomes, where the set of allowed outcomes is either YES/NO or YES/NO/EXPERT. Note that the highest accuracy is highlighted in bold. By restricting the possible outcomes to YES/NO only, it is evident that the *KR* model outperforms both *ML* and *Hybrid* models. However, when all outcomes are allowed, namely YES/NO/EXPERT, then the *Hybrid* model performs on par with the *KR* model, with both *KR* and *Hybrid* models outperforming the *ML* model. It is worth noting that the results of *KR* and *Hybrid* models, when all three outcomes are allowed, are not directly comparable to the results of the *ML* model, since the ML model does not refer patients to a medical expert.

Finally, Table 8 provides a detailed list of outcomes for misclassified patients (with misclassified outcomes highlighted in bold). It is evident that all eight patients where misclassified by the *ML* model. In comparison, only 3 (out of eight) patients were misclassified by the *KR* model, with additional three patients being classified correctly (in terms of YES/NO outcomes) and two patients referred to a medical expert. In general, our expectation is that with more patients being assessed in the future, a number of patients will be misclassified by the *KR* model while being correctly classified by the *ML* model, and vice versa. Thus, the *Hybrid* model will provide the best overall results since it will misclassify only patients that are misclassified by both *KR* and *ML* models, with referrals to medical expert (due to conflicts between *KR* and *ML* models) serving as valid outcomes.

## Conclusions and future work

In order to introduce automation support to the diagnosis of adult ADHD, we used a combination of AI technologies: a machine learning model that was trained from clinical data of past cases, provided by an NHS Trust, and a knowledge model representing domain knowledge captured from clinical experts. The AI technology takes as input the same clinical data routinely collected by the NHS upon referral, and comes up with three possible outcomes: has ADHD, does not have ADHD, or consult medical expert.

Results obtained so far show great promise of the technology, because it can accurately identify clear-cut cases where a decision can be safely made, while referring the more complex cases for further assessment by a clinical specialist. This approach leads to increased productivity and throughput of cases, significantly reducing waiting lists and speeding up diagnosis and, where needed, treatment.

The combined use of symbolic AI and machine learning has the potential to combine the strengths of both approaches: explainability and easy transferability based on the knowledge model, and adaptability and refinement based on the machine learning model. Generally speaking, we expect the machine learning model to gain a greater role in the future as the data basis is expanded as new cases are treated and their data is collected. Another benefit of having a machine learning model is that it provides input even for cases that are referred to clinical specialists—the latter can conduct their evaluation with the additional information that the AI algorithm has a tendency to a particular outcome.

At present, the ongoing trial is tested in the largest NHS Service for adults with ADHD in the UK, with the developed decision support tool being used by 8 clinicians. In doing so, we are trying, among others, to (a) establish the predictive accuracy; (b) investigate how to best embed the AI technology in clinical practice by defining relevant clinical pathways, and (c) establishing economic value. For the latter, one important parameter to determine is what proportion of cases can be handled automatically, and what percentage will need consultation of a human expert. Alternatively, the difficult cases will be dealt with by senior clinical experts, while the clear-cut cases will receive a recommended diagnosis by the AI algorithm which will be verified by a less senior clinician.

It would also be interesting to investigate the use of alternative approaches to constructing the predictive model, e.g., using the recently proposed fuzzy rule-based models like the ones proposed in [35, 19], which may work better while dealing with the uncertainty and linguistic imprecision embedded in the cognitive tests, as well as other interpretable models such as Naive Bayes that is able to explicitly demonstrate the contributions made by each attribute towards the overall decision.

## Footnotes

1 https://www.nashia.org/pdf/hotopics/pa-helps-screening-tool.pdf

## References

[1] Thapar A, Cooper M. Attention deficit hyperactivity disorder. The Lancet. 2016;387(10024):1240. 10.1016/S0140-6736(15)00238-X. http://www.sciencedirect.com/science/article/pii/S014067361500238X. 10.1016/S0140-6736(15)00238-X 26386541

[2] American Psychiatric Association (2013). Diagnostic and Statistical Manual of Mental Disorders (DSM-5).

[3] Asherson P, Buitelaar J, Faraone S, Rohde L (2016). Adult attention-deficit hyperactivity disorder: key conceptual issues. Lancet Psychiatry.

[4] National Institute for Health and Care Excellence (NICE) (2018). Attention deficit hyperactivity disorder: diagnosis and management [NG87].

[5] Fields SA, Johnson WM, Hassig MB (2017). Adult ADHD: addressing a unique set of challenges. J Fam Pract..

[6] Spencer T, Biederman J, Wilens T, Faraone S, Prince J, Gerard K, Doyle R, Parekh A, Kagan J, Bearman SK (2001). Efficacy of a mixed amphetamine salts compound in adults with attention-deficit/hyperactivity disorder. Arch General Psychiatry.

[7] Arnold L, Hodgkins P, Kahle J, Madhoo M, Kewley G (2015). Long-term outcomes of ADHD: academic achievement and performance. J Atten Disord..

[8] Loe IM, Feldman HM (2007). Academic and educational outcomes of children with ADHD. J Pediatric Psychol..

[9] Langberg JM, Molina BS, Arnold LE, Epstein JN, Altaye M, Hinshaw SP, Swanson JM, Wigal T, Hechtman L (2011). Patterns and predictors of adolescent academic achievement and performance in a sample of children with attention-deficit/hyperactivity disorder. J Clin Child Adolesc Psychol..

[10] Cook J, Knight E, Hume I, Qureshi A (2014). The self-esteem of adults diagnosed with attention-deficit/hyperactivity disorder (ADHD): a systematic review of the literature. ADHD Atten Deficit Hyperact Disord..

[11] Adamou M, Arif M, Asherson P, Aw TC, Bolea B, Coghill D, Gujónsson G, Halmøy A, Hodgkins P, Müller U (2013). Occupational issues of adults with ADHD. BMC Psychiatry.

[12] Dalsgaard S, Østergaard SD, Leckman JF, Mortensen PB, Pedersen MG (2015). Mortality in children, adolescents, and adults with attention deficit hyperactivity disorder: a nationwide cohort study. The Lancet.

[13] Vibert S (2018). Your attention please: the social and economical impact of ADHD.

[14] Audit support (adults) [CG72]. National Institute for Health and Clinical Excellence, Attention deficit hyperactivity disorder (ADHD), (2008)

[15] Asherson P, Adamou M, Bolea B, Muller U, Morua SD, Pitts M, Thome J, Young S (2010). Is ADHD a valid diagnosis in adults? Yes. Bmj..

[16] Chen T, Su P, Shang C, Hill R, Zhang H, Shen Q. Sentiment Classification of Drug Reviews Using Fuzzy-rough Feature Selection. In: *2019 IEEE International Conference on Fuzzy Systems*, pp. 1–6 (IEEE, 2019).

[17] Su P, Zhao Y, Chen T, Xie J, Zhao Y, Qi H, Zheng Y. Exploiting Reliability-Guided Aggregation for the Assessment of Curvilinear Structure Tortuosity. In *International Conference on Medical Image Computing and Computer-Assisted Intervention*, pp. 12–20 (Springer, 2019).

[18] Chen T, Shang C, Su P, Antoniou G, Shen Q. Effective diagnosis of diabetes with a decision tree-initialised neuro-fuzzy approach. In *UK Workshop on Computational Intelligence*, pp. 227–239 (Springer, 2018)

[19] Chen T, Shang C, Su P, Shen Q (2018). Induction of accurate and interpretable fuzzy rules from preliminary crisp representation. Knowl-Based Syst..

[20] Chen T, Antoniou G, Adamou M, Tachmazidis I, Su P (2020). Automatic diagnosis of attention deficit hyperactivity disorder using machine learning. Appl Artif Intell..

[21] Conners CK, Erhardt D, Sparrow EP (1999). Conners’ adult ADHD rating scales (CAARS): technical manual.

[22] Skinner HA (1982). The drug abuse screening test. Addict Behav..

[23] Langbehn DR, Pfohl BM, Reynolds S, Clark LA, Battaglia M, Bellodi L, Cadoret R, Grove W, Pilkonis P, Links P (1999). The Iowa personality disorder screen: development and preliminary validation of a brief screening interview. J Personal Disord..

[24] Saunders JB, Aasland OG, Babor TF, De la Fuente JR, Grant M (1993). Development of the alcohol use disorders identification test (AUDIT): WHO collaborative project on early detection of persons with harmful alcohol consumption-II. Addiction.

[25] Hirschfeld RM (2002). The Mood Disorder Questionnaire: a simple, patient-rated screening instrument for bipolar disorder. Prim Care Companion J Clin Psychiatry.

[26] Spitzer RL, Kroenke K, Williams JB, Löwe B (2006). A brief measure for assessing generalized anxiety disorder: the GAD-7. Arch Intern Med..

[27] Picard M, Scarisbrick D, Paluck R (1991). HELPS: a brief screening device for traumatic brain injury.

[28] Ramos-Quiroga JA, Nasillo V, Richarte V, Corrales M, Palma F, Ibáñez P, Michelsen M, Van de Glind G, Casas M, Kooij JS (2019). Criteria and concurrent validity of DIVA 2.0: a semi-structured diagnostic interview for adult ADHD. J Atten Disord.

[29] Morgan S (2000). Clinical risk management: a clinical tool and practitioner manual.

[30] Reh V, Schmidt M, Lam L, Schimmelmann BG, Hebebrand J, Rief W, Christiansen H (2015). Behavioral assessment of core ADHD symptoms using the QbTest. J Atten Disord..

[31] Chen T, Shang C, Yang J, Li F, Shen Q (2019). A new approach for transformation-based fuzzy rule interpolation. IEEE Trans Fuzzy Syst..

[32] Wu X, Kumar V, Quinlan JR, Ghosh J, Yang Q, Motoda H, McLachlan GJ, Ng A, Liu B, Philip SY (2008). Top 10 algorithms in data mining. Knowl Inf Syst..

[33] Hosmer DW, Lemeshow S, Sturdivant RX (2013). Applied logistic regression.

[34] Liaw A, Wiener M (2002). Classification and regression by randomForest. R News..

[35] Chen T, Shen Q, Su P, Shang C (2016). Fuzzy rule weight modification with particle swarm optimisation. Soft Comput..

